# Supplementary figures and images for: Hate speech detection and racial bias mitigation in social media based on BERT model
Source: PLoS One. 2020 Aug 27;15(8):e0237861. doi: 10.1371/journal.pone.0237861 (PMC7451563; doi:10.1371/journal.pone.0237861)

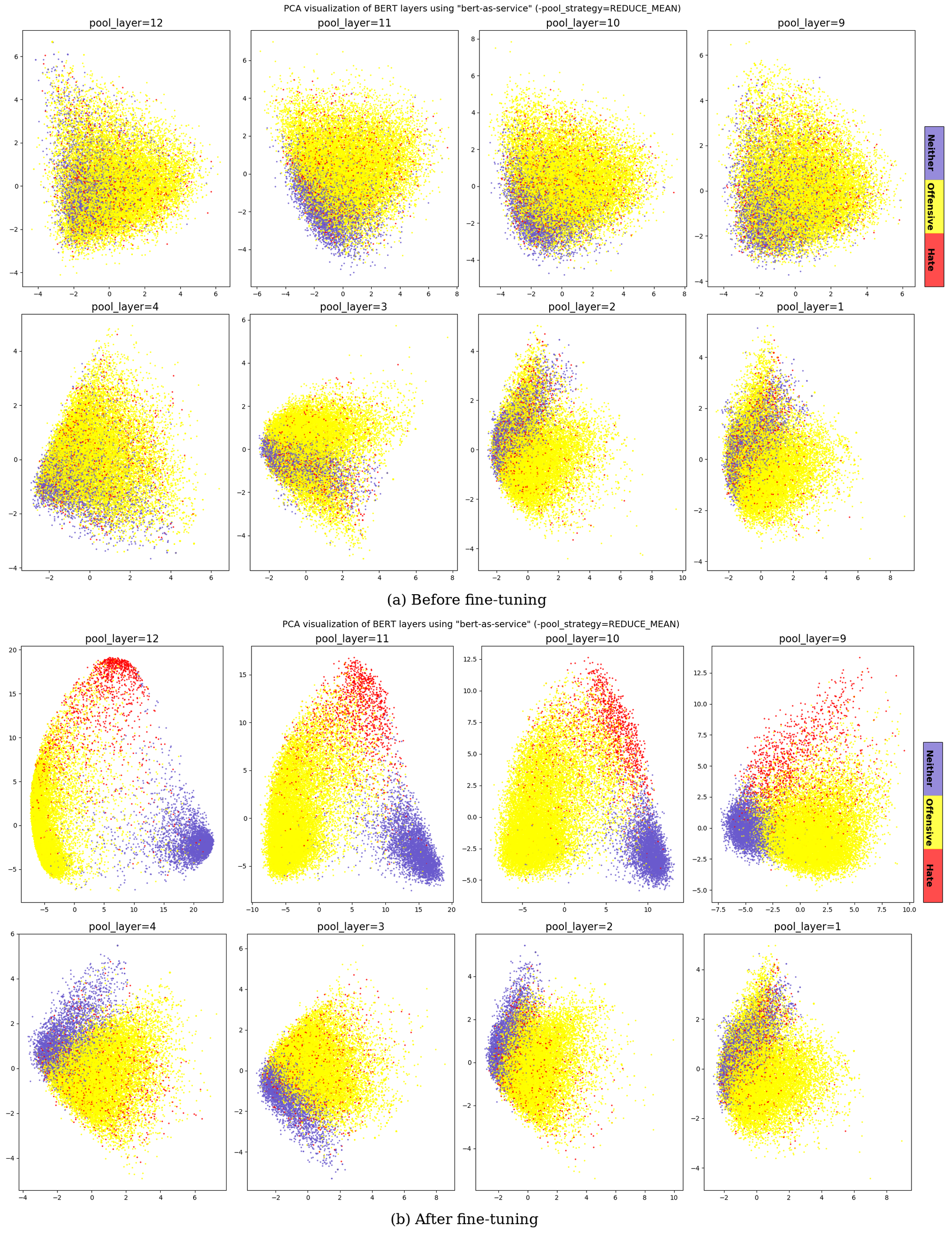

Supplement: S1 Fig — (TIF) [file pone.0237861.s001.tif]

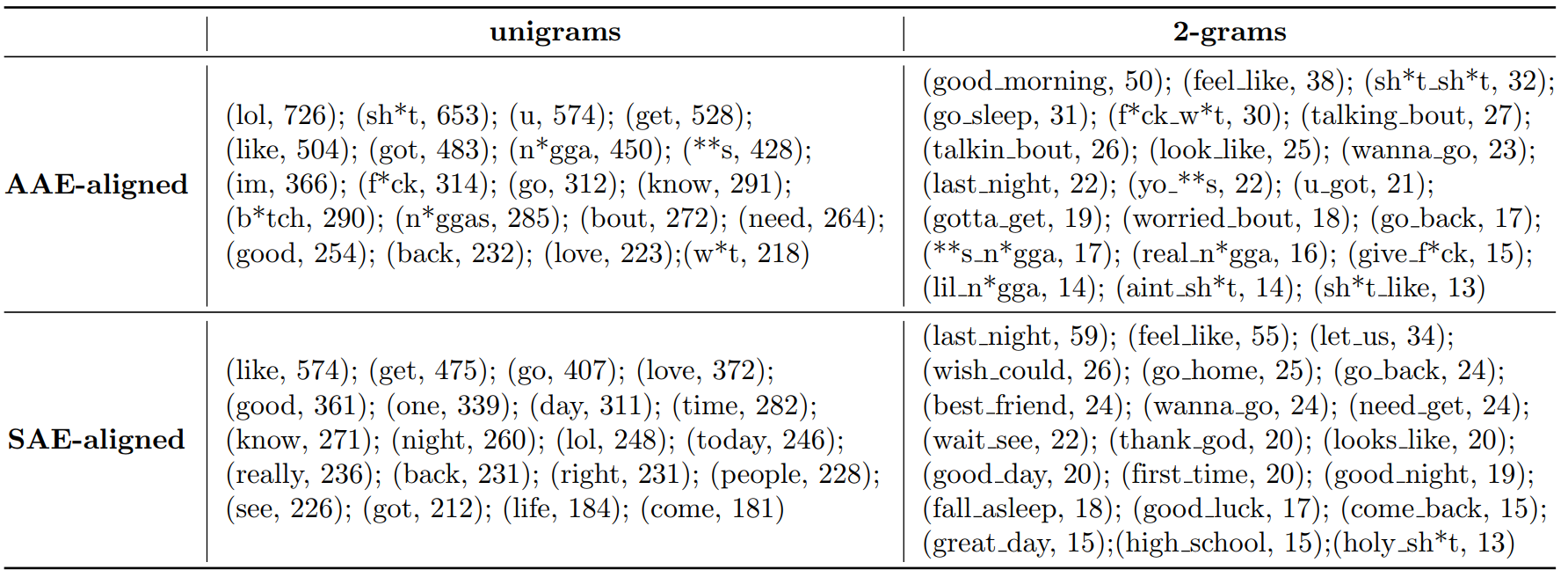

Supplement: S1 Table — Extracting unigrams and 2-grams that occur most frequently in tweets written by AAE and SAE groups, shows that some particular phrases such as “n*gga”, “b*tch”, “sh*t”, “f*ck_w*t”, “**s_n*gga”, etc., are common in AAE dialects and are highly correlated with negative classes (Racism, Sexism, Hate and Offensive) in hate and offensive datasets. (TIF) [file pone.0237861.s002.tif]
